# Effects of Cardamom on Neuroinflammation, Learning and Memory in Mice Fed a Cafeteria Diet

**DOI:** 10.1002/edm2.70130

**Published:** 2026-01-31

**Authors:** Anfal AL‐Dalaeen, Nour Batarseh, Sally Atawneh

**Affiliations:** ^1^ Department of Clinical Nutrition and Dietetics, Faculty of Allied Medical Sciences Applied Science Private University Amman Jordan; ^2^ Department of Clinical Pharmacy Therapeutics, Faculty of Pharmacy Applied Science Private University Aqaba Jordan

**Keywords:** brain function, cardamom, memory, neuro‐inflammation, obesity

## Abstract

**Background:**

The consumption of a cafeteria diet is described as deleterious to cognitive performance, potentially due to inducing inflammation in the brain. Cardamom, a potent antioxidant, may benefit brain health. In the current study, we assessed the effects of a cafeteria diet on neuroinflammation and its reversal by dietary cardamom.

**Methods:**

Thirty‐six male C57BL/6 mice were fed with a cafeteria diet (CAF) to induce obesity for ten weeks. They were then divided into four treatment groups: standard diet (SD), cafeteria diet (CAF), cafeteria diet with cardamom (CAF‐CARD) and standard diet with cardamom (SD‐CARD). After administering cardamom orally (500 mg/kg/day) for 4 weeks, the mice were subjected at week 14 to behavioural tests assessing learning and memory, and hippocampal tumour necrosis factor (TNF‐α) levels were measured to evaluate neuroinflammation.

**Results:**

The TNF‐alpha level in the CAF group was higher than in the SD group (*p* < 0.001), and significantly lower in the CAF‐CARD group compared to the CAF group (*p* < 0.01). The recognition index (RI) was significantly lower in the CAF group, while cardamom supplementation improved the RI compared to the CAF group (*p* < 0.01). There was a significant difference in spatial memory between SD and CAF groups (*p* < 0.01). In terms of digging behaviour, which indicates anxiety, mice in the CAF group buried 58% of the marbles compared to 38% in the SD group (*p* < 0.01). However, this behaviour decreased in the CAF‐CARD group compared to the CAF group (*p* < 0.001).

**Conclusion:**

Cardamom appears to be beneficial for obesity‐related cognitive impairments and dysfunction in the hippocampus.

## Graphical abstract

Cardamom supplementation (500 mg/kg/day) improved memory, reduced anxiety and neuroinflammation in mice fed with a cafeteria diet. Behavioural tests showed enhanced cognitive functions, while TNF‐α and lipid markers decreased significantly. These findings suggest cardamom's potential as a neuroprotective agent against diet‐induced memory and metabolic dysfunction.

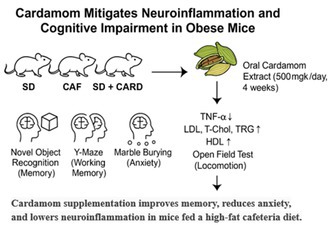

## 1 Introduction

Cardamom ( *Elettaria cardamomum* ) is a spice known for its aromatic properties and potential health benefits, including antioxidant and anti‐inflammatory effects [1]. As a nutraceutical, cardamom is rich in polyphenols and terpenoids, which have been reported to positively influence mood, behaviour and metabolism [2, 3].

Studies in both human and animal models have highlighted a wide range of health benefits resulting from cardamom consumption, including improvements in metabolic function, reductions in dyslipidemia and body weight, regulation of blood pressure and prevention of obesity [1, 4, 5]. In mice fed a cafeteria (CAF) diet high in carbohydrates and fats, cardamom intake has been shown to improve glucose tolerance, enhance insulin sensitivity and mitigate oxidative stress [6, 7]. The CAF diet is known to contribute to neuroinflammation by disrupting the blood–brain barrier and increasing pro‐inflammatory cytokines like TNF‐α and IL‐6 [8, 9]. Similar outcomes have been observed in diabetic mice and those administered dexamethasone [10]. Additionally, mice consuming a diet enriched with cardamom exhibited a reduced atherogenic index. Cardamom also demonstrated the ability to regulate blood pressure in mice [11] and prevent obesity [4].

The health benefits of cardamom are largely attributed to its antioxidant and anti‐inflammatory properties. Its polyphenols and flavonoids neutralise reactive oxygen species (ROS), thereby reducing oxidative stress and protecting tissues from damage that can lead to accelerated aging, insulin resistance and neurodegenerative diseases [2, 12, 13]. Specific cardamom polyphenol compounds, such as 1,8‐cineole and 1,6,10‐dodecatrien‐3‐ol, have been shown to reduce fat mass, promote vasodilation and provide both anti‐inflammatory and antioxidant benefits [14].

Cardamom also enhances antioxidant defence by increasing the activity of glutathione, catalase and superoxide dismutase, thereby protecting neuronal cells from lipid peroxidation [15, 16]. Furthermore, cardamom influences key intracellular signalling pathways, modulating acetylcholinesterase activity, stimulating neurotrophic factors such as brain‐derived neurotrophic factor (BDNF) and reducing amyloid‐β aggregation in the hippocampus and cortex, which collectively support memory, cognitive function and neuroprotection [2, 17].

Despite these promising mechanisms, research directly examining the effects of cardamom on learning, memory and neuroinflammation remains limited. We hypothesised that cardamom supplementation would reduce neuroinflammation and improve cognitive performance in mice fed a cafeteria diet, as measured by TNF‐α levels and behavioural tests assessing memory and anxiety. This study aims to explore the neuroprotective potential of cardamom in the context of diet‐induced obesity and hippocampal inflammation.

## 2 Materials and Methods

### 2.1 Experimental Design

Thirty‐six 6‐week‐old C57BL/6 male mice (20 ± 0.1 g), were acquired from Jordan University of Science and Technology, located in Irbid, Jordan. Following a 1‐week acclimatisation period, the mice were housed in cages at 22°C with humidity levels between 35% and 70%, under a 12‐h light/dark cycle. Tap water and a standard pellet diet were available ad libitum. The experiment comprised two groups: the standard diet (SD) group which received a standard diet (SD) consisting of 20% fat, 25% protein and 55% carbohydrate LabDiet (NIH‐31), and a cafeteria diet (CAF) group which received a diet comprising 38.1% fat, 16.6% protein and 35.5% carbohydrate for 10 weeks as described in Table S1.

**FIGURE 1:**
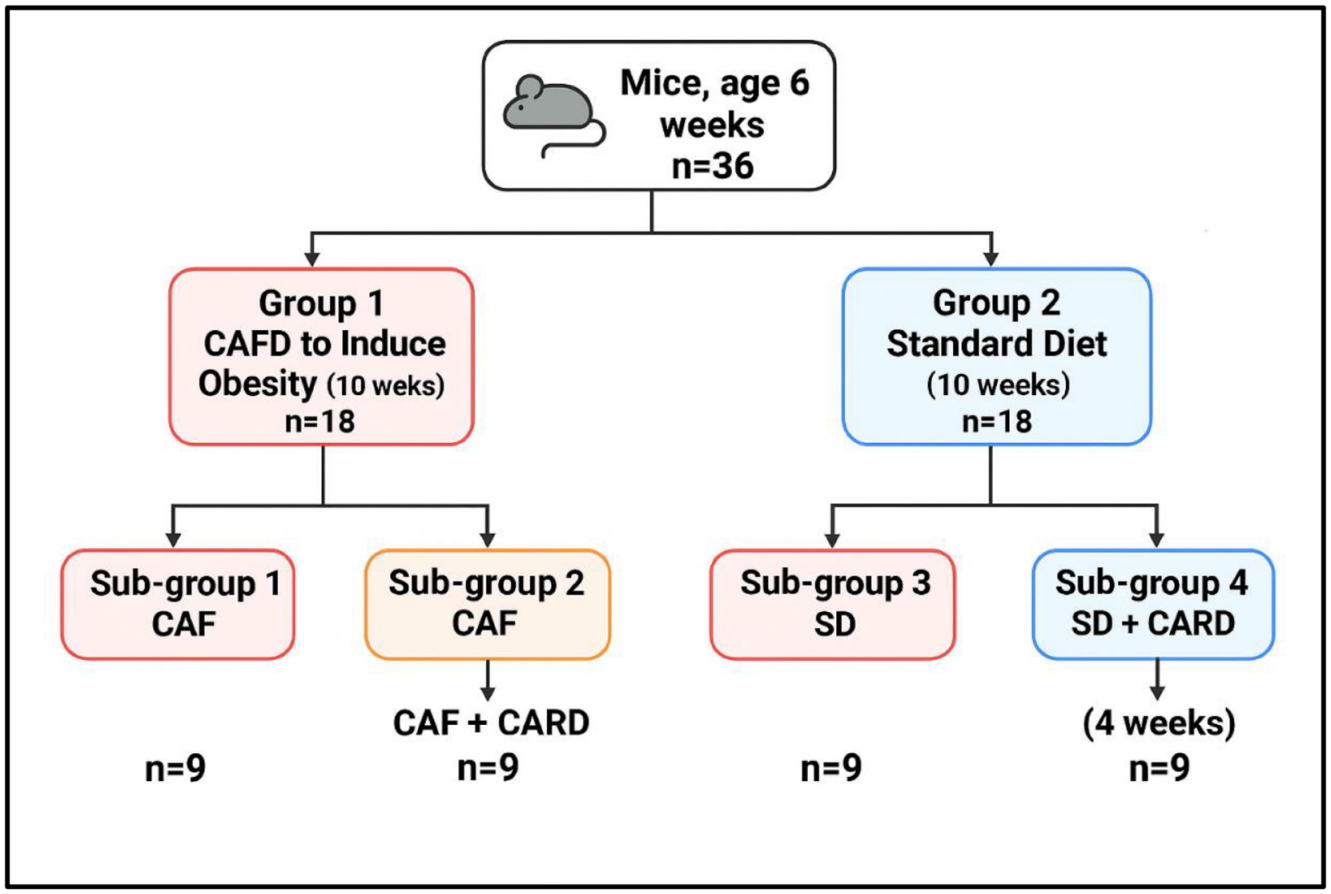
Shows the experiment design of the study. Group 1 was fed with a cafeteria diet to induce obesity for 10 weeks. Group 2 was used as a standard diet group. All groups were divided into four subgroups where subgroup (1) continued on the cafeteria diet, subgroup (2) continued on the cafeteria diet with administered orally cardamom extract 500 mg/kg, subgroup (3) continued on a standard diet and finally subgroup (3) continued on a standard diet with cardamom extract 500 mg/kg. CAF, cafeteria diet.

Following the 10‐week induction phase with a cafeteria diet, mice were randomly assigned to four treatment groups (*n* = 9 per group): CAF, CAF‐CARD, SD and SD‐CARD. Cardamom extract was administered orally at a dose of 500 mg/kg/day for 4 weeks, beginning immediately after the induction phase. The treatment was given once daily via oral gavage. This regimen was designed to evaluate the effects of cardamom on neuroinflammation and cognitive function as shown in Figure 1.

To assess lipid profile, blood samples were collected from retro‐orbital veins. Furthermore, the hippocampus was harvested for the measurement of TNF‐α. All procedures involving animals received approval from the Ethics Committee of Applied Science Private University.

### 2.2 Obesogenic Mice Diet

The energy content of the standard diet is 3.57 kcal/g, derived from 67% carbohydrates, 24% proteins and 9% fats, as shown in Table 1. The cafeteria diet was formulated to mimic the diversity and energy density characteristic of a human obesogenic diet, incorporating commercial snacks with 4.84 kcal/g, which consist of 35.3% carbohydrates, 37.1% fats and 17.6% proteins [10]. Additionally, a 10% sucrose solution was offered alongside clean water for 10 weeks. Food intake was recorded daily with corrections for any spillage to ensure accurate consumption assessment. Body weight was measured weekly using a digital balance. Comprehensive nutritional details of the cafeteria diets are provided in Table 1 and were based on formulations used in previous studies to induce obesity [18, 19].

**TABLE 1:** Serum lipid profile and hippocampal inflammation markers in mice across dietary and treatment groups.

Variable	SD	CAF	SD‐CARD	CAF‐CARD
LDL (mg/dL)	53.30 ± 11.94*^a^	120.03 ± 24.54*^b^	60 ± 12.94*^c^	110.027 ± 24.44
T‐Chol (mg/dL)	62.13 ± 10.61*^a^	113.18 ± 22.42*^b^	65.03 ± 11.61*^c^	100.48 ± 21.42
HDL (mg/dL)	46 ± 6.16*^a^	73.41 ± 11.54*^b^	55 ± 6.0	61.43 ± 12.52
TRG (mg/dL)	60.31 ± 17.80	85 ± 21.01*^b^	60 ± 10.54	61 ± 11.00
FBG (mg/dL)	100 ± 11.4	106 ± 12.43	90 ± 11.8	97 ± 15.0
Atherogenic index^a^	0.28 ± 0.10*^a^	0.53 ± 0.16*^b^	0.29 ± 0.12*^c^	0.43 ± 0.10
TNF‐α (ng/L)	82.89 ± 20.14*^a^	112.37 ± 11.54*^b^	70 ± 11.14*^c^	98 ± 12.17

*Note:* Data are presented as means (with standard deviations) and are considered significant at (*p* < 0.05) under the following conditions: (*a) when compared to SD with CAF, (*b) when compared to SD with CAF‐CAFD and (*c) when compared to CAF‐CARD.

Abbreviations: CAF, cafeteria diet; CAF‐CARD, cafeteria diet with cardamom; FBG, fasting blood glucose; HDL, high‐density lipoprotein; LDL, low‐density lipoprotein; SD, standard diet; SD‐CARD, standard diet with cardamom; T‐Chol, total cholesterol; TRG, triglyceride.

^a^ Atherogenic index: cholesterol‐HDL‐C/HDL‐C.

### 2.3 Cardamom Water Extract

**FIGURE 2:**
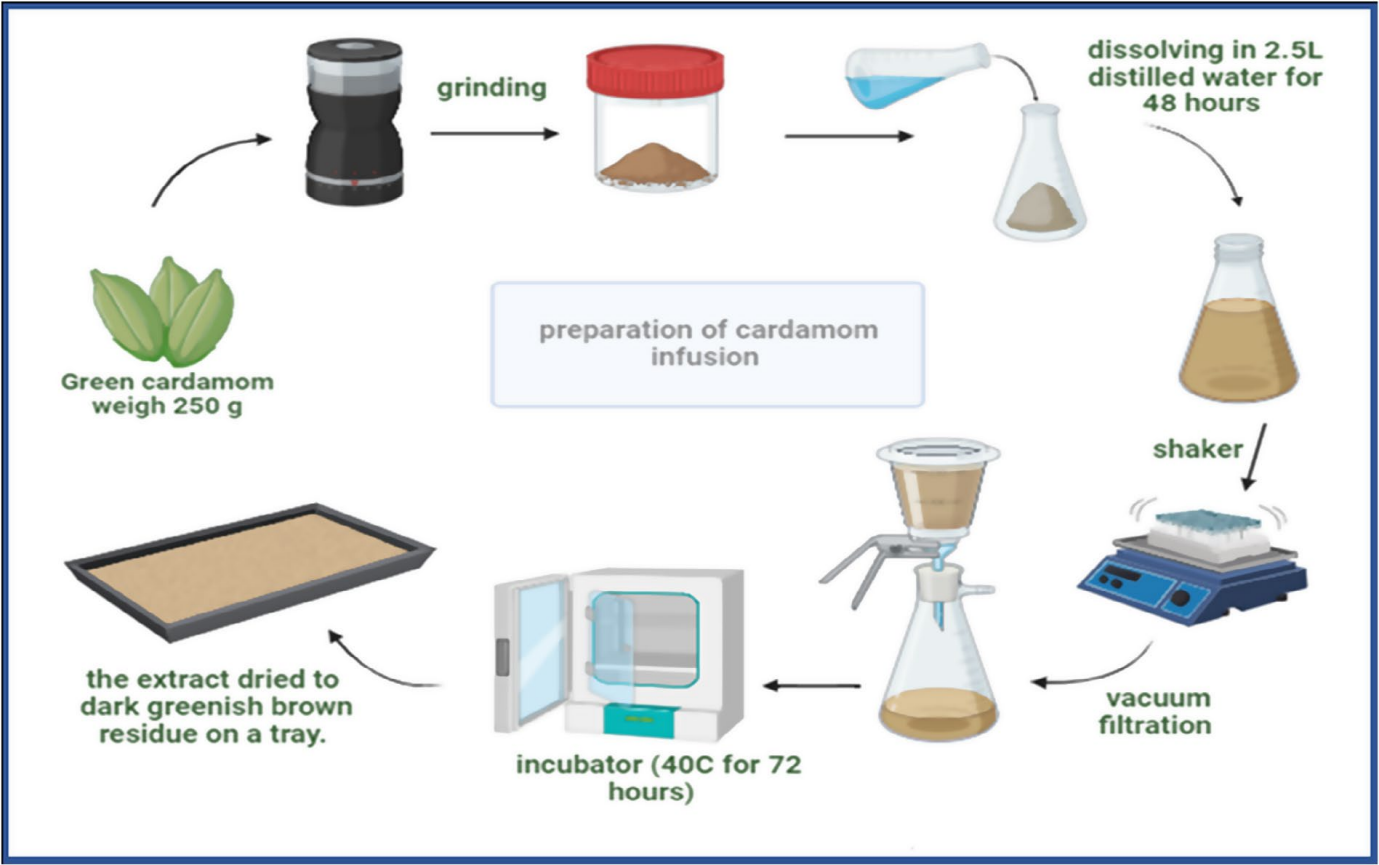
Preparation process of cardamom extract.

Seeds of cardamom were purchased locally in Amman, Jordan. To prepare the cardamom water extract, the dried seeds were ground into powder, and 250 g of powder was soaked in 2.5 L of distilled water for 48 h. The extract was filtered, concentrated under reduced pressure and dried in an incubator at 40°C for 72 h to yield a dark greenish‐brown residue. The extract was standardised to contain 500 mg/kg of active substance [20] as shown in Figure 2.

### 2.4 Intraperitoneal Glucose Tolerance Test (IPGTT)

After a 10‐week CAF diet, the IPGTT was performed after fasting for 12 h, and glucose was injected into mice at 2 g/kg of body weight [21]. Blood glucose was measured at zero time and 30, 60 and 120 min to assess the blood glucose tolerance.

### 2.5 Measurements of Serum Lipid Profile and Inflammatory Markers in the Hippocampus

To assess total cholesterol (T‐Chol), low‐density lipoproteins (LDL), high‐density lipoproteins (HDL) and triglycerides (TRG) in plasma, enzymatic colorimetric assays with commercial kits were used. Blood samples were collected after a 12‐h fasting period, consistent with the protocol used for the IPGTT. The TNF‐α levels in the hippocampus were measured using the TNF‐α ELISA Kit (Invitrogen, BMS607‐3TEN), following the manufacturer's instructions. All samples were analysed in duplicate to ensure the reliability and reproducibility of the results.

### 2.6 Behavioural Analysis

#### 2.6.1 Open Field Test and Novel Object Recognition (NOR) Test

The open field test was used to assess locomotor activity and anxiety‐like behaviours. Mice were placed individually in a square arena (40 cm × 40 cm) with high walls, and their movement was recorded for 5 min using a video tracking system. The time spent and distance travelled in the centre versus periphery were analysed [22]. The NOR behavioural test was conducted for two consecutive days. On day one (training session), mice were placed in the arena with two identical objects and allowed to explore for 5 min. On day two (test session), one object was replaced with a novel object, and exploration time was recorded [23].

#### 2.6.2 Y‐Maze Test

The Y‐maze test was used to assess working memory. The apparatus consisted of three arms (each 35 cm long, 5 cm wide and 15 cm high) arranged in a Y‐shape. Mice were placed in the centre and allowed to discove the box freely for 10 min. The sequence and number of arm entries were recorded, and spontaneous alternation behaviour was calculated [24].

#### 2.6.3 Marble Burying Test

The marble burying test was used to assess anxiety and repetitive behaviour. Fifty glass marbles were evenly spaced on 5 cm deep bedding in a standard cage. Mice were placed in the cage for 30 min, and the number of marbles buried (to at least two‐thirds depth) was counted [25].

### 2.7 Statistical Analysis

Data were analysed using SPSS v27.0 (Chicago, IL, USA). Two‐way ANOVA was used to assess the effects of diet, treatment and their interaction. Repeated measures ANOVA was applied for body weight and IPGTT, with AUC calculated for IPGTT. Tukey's post hoc test was used for multiple comparisons. Significance was set at *p* < 0.05. Full statistical results, including *F*‐values, degrees of freedom and *p* values for main effects and interactions, are provided in Table S2.

## 3 Result

**FIGURE 3:**
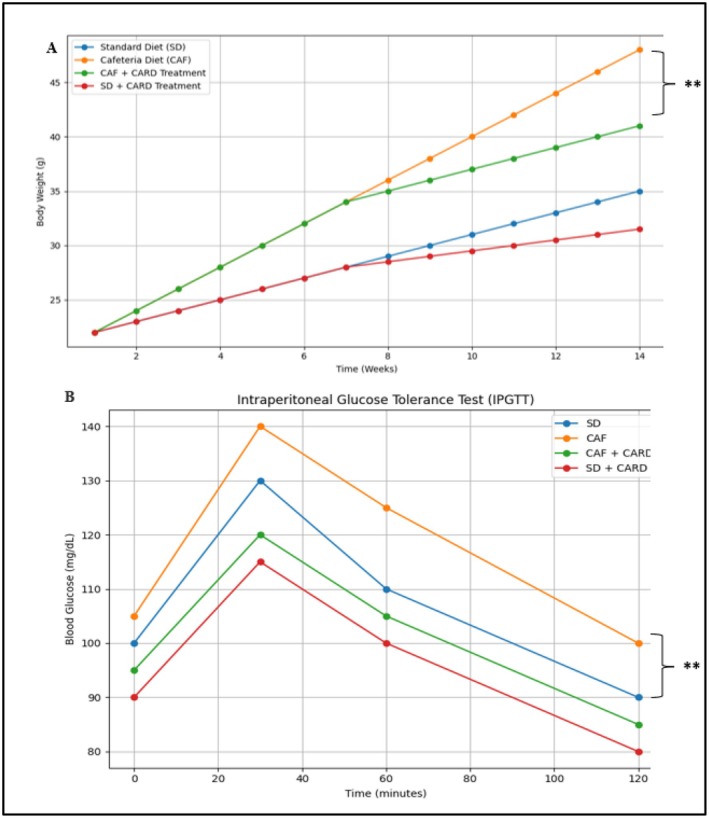
Scatter plot showing changes in body weight (Δ Final − Baseline) across four groups: SD, SD‐CARD, CAF and CAF‐CARD. Data are mean ± SD (*n* = 9). Asterisks indicate significant differences between groups at the final time point only, based on Tukey's post hoc test (**p < 0.01).

Figure 3 illustrates the effects of a cafeteria diet and cardamom supplementation on weight gain and blood glucose levels in mice. In Figure 3A, mice on the CAF showed the highest weight gain over 14 weeks (*p* < 0.01). The CAF‐CARD significantly reduced this weight gain compared to the CAF group (*p* < 0.01). In Figure 3B, blood glucose levels peaked at 30 min for all groups except the SD‐CARD, which remained stable. The CAF group had significantly higher glucose levels at 30 min compared to the SD group (*p* < 0.01), while CAF‐CARD significantly lowered these levels (*p* < 0.01).

**FIGURE 4:**
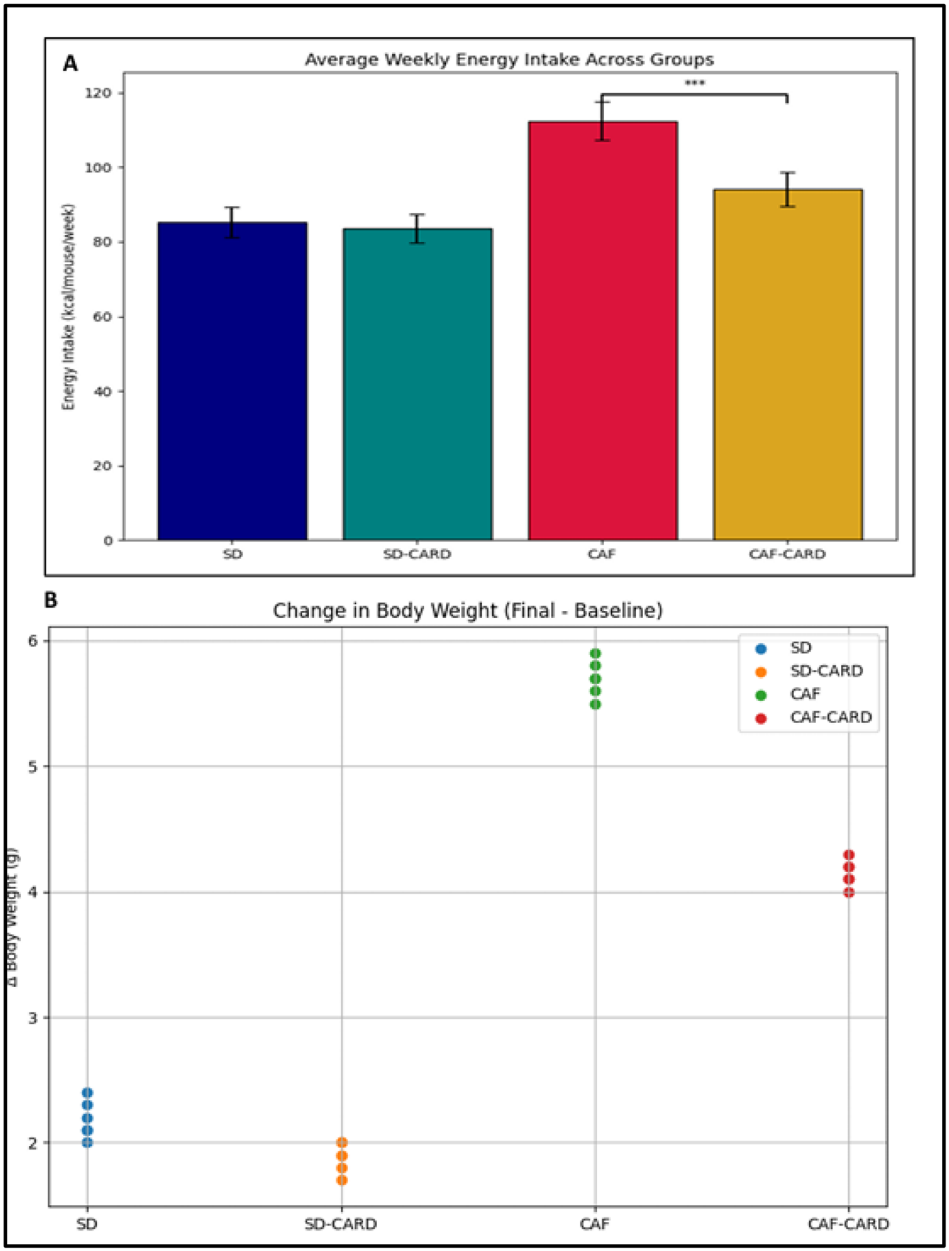
Scatter plot depicting individual changes in body weight (Δ Body Weight; Final − Baseline) across four experimental groups: standard diet (SD), standard diet with cardamom intervention (SD‐CARD), cafeteria diet (CAF) and cafeteria diet with cardamom intervention (CAF‐CARD). Data are means ± SD for nine mice/group. ***Mean *p* value < 0.001.

As shown in Figure 4, changes in body weight (Δ Body Weight; Final − Baseline) varied significantly across the four experimental groups. Animals in the CAF and CAF‐CARD groups exhibited greater increases in body weight compared to those in the SD and SD‐CARD groups. Notably, the CAF‐CARD group showed a statistically significant difference in weight gain relative to the SD group (*p* < 0.05).

Table 1 shows T‐Chol and TRG plasma levels for all mice. The T‐Chol and TRG plasma concentrations were increased in mice fed a CAF diet as compared to an SD group (*p* < 0.01). Notably, the addition of cardamom significantly lowered the levels of T‐Chol and TRG in mice in the SD‐CAD group and CAF‐CARD groups (*p* < 0.05). Furthermore, LDL and T‐Chol levels were also found to be higher in the CAF diet group relative to the SD group (*p* < 0.01), but this was subsequently reduced with cardamom supplementation (Table 1). In contrast, HDL concentrations increased in both the CAF and CAF‐CARD groups that received cardamom powder. To evaluate inflammation, TNF‐α levels were assessed in the hippocampus. The concentration of TNF‐α was significantly elevated in the CAF diet group compared to the standard mice (*p* < 0.01). Conversely, a significant reduction in TNF‐α levels (p < 0.05) was found in CAF compared to the CAF‐CARD group (112.36 ± 11.53 vs. 98 ± 13.16; *p* < 0.01; respectively).

**FIGURE 5:**
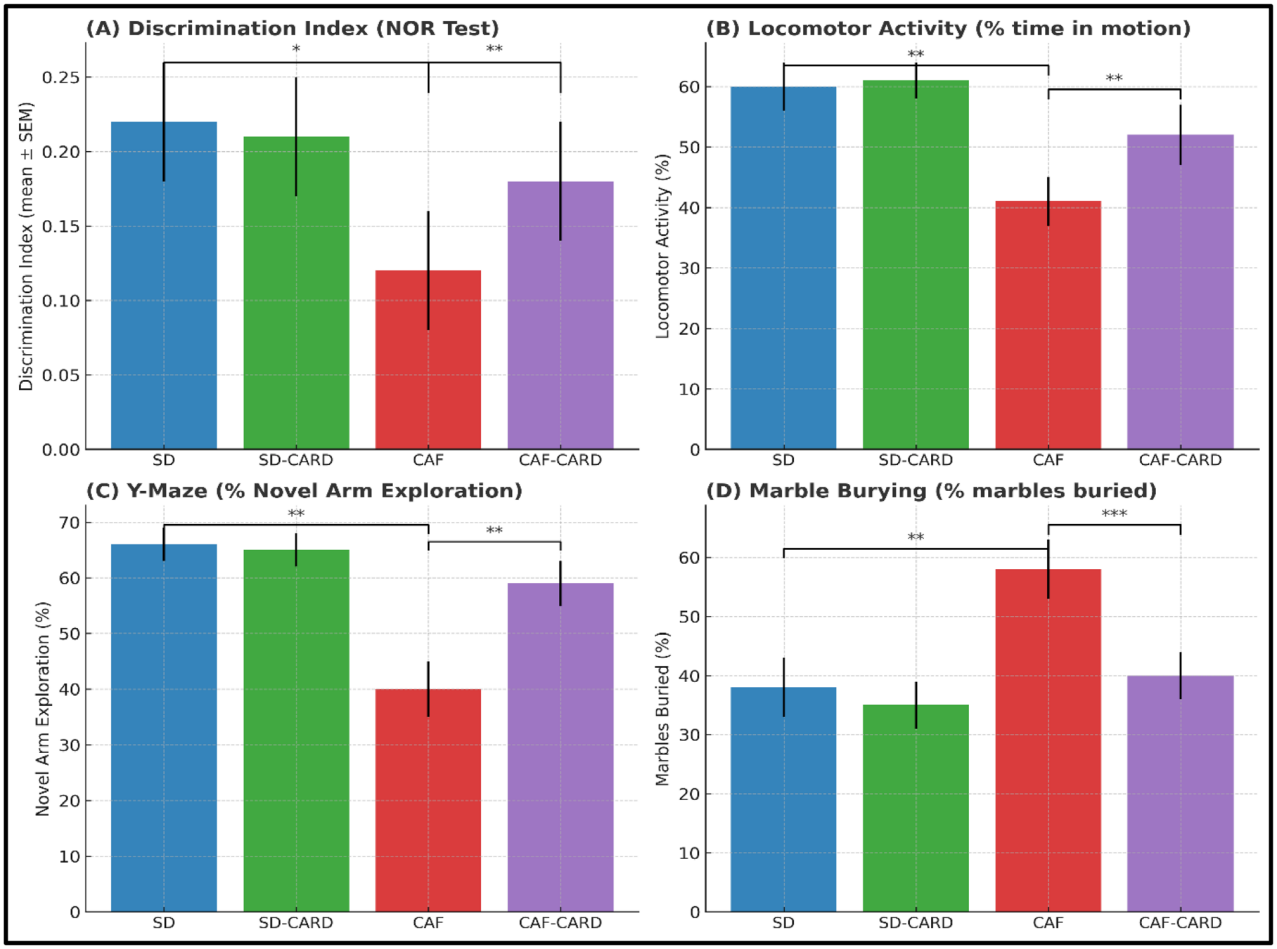
Effects of cardamom on different behaviour tests. (A) Time required to interact with the familiar vs. novel object during testing, which is indexed as discrimination index, is computed using the formula (T_N − T_F)/(T_N + T_F), where T_N represents the duration spent with the novel object, and T_F denotes the duration spent with the familiar object. (B) The locomotor activity of the animals during an open field test was recorded. The data was presented as a percentage of the time the animals were in motion relative to the total duration of the test. (C) Percentage of time allocated to the novel arm, starting arm and other arm; (D) Marble burying behaviour as a percent. The values are presented as mean ± SEM of nine animals per group. The data were analysed by Student's *t*‐test. *Mean *p* < 0.05, **Mean *p* value < 0.01,***Mean *p* < 0.001.

Figure 5 presents the behavioural test across experimental groups. In the NOR test, the DI in the CAF group was lower than of the SD group (0.12 ± 0.04 vs. 0.22 ± 0.084; *p* < 0.05), suggesting an impairment in recognition memory. Importantly, cardamom supplementation in the CAF‐CARD group resulted in a significant improvement in DI compared to the CAF group (0.18 ± 0.04 vs. 0.11 ± 0.026; *p* < 0.01), as shown in Figure 5A.

The cafeteria diet (CAF) group exhibited marked impairments in behaviour and cognition, including reduced locomotor activity (mean distance ~10 m, *p* < 0.01), a 40% decrease in novel arm exploration (*p* < 0.01) and heightened anxiety‐related behaviour (*p* < 0.001). Notably, cardamom supplementation significantly ameliorated these deficits. Compared to the CAF group, CAF + CARD mice demonstrated increased activity levels (*p* < 0.01), enhanced working memory and exploration (*p* < 0.01) and reduced anxiety‐related behaviours (40% vs. 58%, *p* < 0.001). Collectively, these findings indicate that cardamom supplementation can counteract the adverse cognitive and behavioural effects of an unhealthy diet, underscoring its potential role in supporting brain health and cognitive function.

## 4 Discussion

Medicinal plants are highly valued for their rich bioactive compounds, particularly polyphenols and flavonoids, which are generally safe and possess antioxidant, antimicrobial, anti‐inflammatory, cardioprotective, immunomodulating and neuroprotective properties. These attributes make them essential for combating infections, wound healing and chronic diseases [26, 27].

*Elettaria cardamomum* , a member of the Zingiberaceae family, is known as the ‘Queen of Spices’ due to its strong and distinctive aroma [28]. Cardamom contains polyphenols and terpenoids that have demonstrated many beneficial health‐related effects on the endocrine and cardiovascular systems, and these effects are achieved through mechanisms such as energy homeostasis, modulation of neural circuits in the hypothalamus, and fatty acid oxidation [2]. It also exerts antihypertensive and antithrombotic effects by restoring nitric oxide levels, reducing inflammatory mediators and inhibiting platelet aggregation [11].

Consumption of a high‐fat diet, such as the CAF diet, is a major risk factor for chronic diseases, including cardiovascular disease and certain types of cancer (e.g., breast, gastric, intestinal and liver) [29], neural disorders [30] and depressive and anxious behaviours [31]. The CAF diet is a major contributor to the development of psychological disorders such as anxiety and depression [32], as well as impaired memory and spatial learning [22]. On the other hand, cardamom shows promise in addressing obesity‐induced metabolic syndrome and diabetes by activating lipoprotein lipase, enhancing cholesterol degradation and reducing lipid absorption from the intestine [28, 33]. Additionally, it enhances memory retention and learning by elevating the levels of dopamine, serotonin and glutathione [34].

Previous studies have demonstrated that cardamom supplementation ameliorates glucose intolerance and lipid abnormalities in rodents fed a high‐carbohydrate, high‐fat diet, attributed to its anti‐inflammatory and antioxidant constituents such as 1,8‐cineole, epicatechin, vanillin, α‐terpineol and ellagic acid [35, 36]. In our study, mice fed a CAF diet for 9 weeks exhibited obesity, glucose intolerance and hyperlipidemia, consistent with prior findings [35]. Winarsi et al. [36] demonstrateda considerable reduction in blood glucose and cholesterol levels in a group of rats treated with 120 mg/kg of cardamom leaf extract for 14 days, where blood glucose and cholesterol levels decreased from 202 to 103 mg/day; and 78 to 56 mg/respectively, suggesting that cardamom may offer protection against diet‐induced obesity‐related metabolic syndrome [36].

In our recent study, we observed that mice fed a CAF diet for 9 weeks showed signs of obesity, glucose intolerance and hyperlipidemia. These effects were confirmed by their increased weight, glucose intolerance and higher blood cholesterol, LDL and triglyceride levels compared to mice fed an SD diet. The findings are similar to previous research that reported experimental animals exposed to a CAF diet for 4–8 weeks developed obesity, hyperlipidemia, glucose intolerance and insulin resistance, where the mechanisms have been linked to mitigating oxidative stress, suppression of antioxidant glutathione, along with elevated inflammatory mediator level in the tissues [18, 37]. Following administration of 500 mg/kg oral cardamom supplementation, body weight, glucose levels and lipid markers significantly improved, indicating that cardamom may benefit individuals with metabolic issues.

Systematic inflammation, neuroinflammation and increased levels of interleukins in the cerebral cortex and hippocampus are key features of the CAF diet [9]. Conversely, cardamom, as a powerful antioxidant, can potentially mitigate inflammation. In a study conducted among adult patients aged 18–75 diagnosed with SARS‐CoV‐2 infection, consuming 500 mg of green cardamom in a pharmaceutical form over 10 days reduced inflammation biomarkers [38]. The mechanisms through which cardamom modulates inflammation include the downregulation of cyclooxygenase‐2 (COX‐2) and iNOS expression and the mitigation of inflammatory cytokines such as interleukin‐6 (IL‐6) and TNF‐α [38] in the colon [39].

A recent study has indicated that supplementation with 200 mg/day of green cardamom combined with a low‐calorie diet reduced TNF‐α levels in the hippocampus following 42 days of treatment [40]. Another clinical trial conducted on 94 women diagnosed with polycystic ovary syndrome reported that combining 3 g of cardamom with a low‐calorie diet over 16 weeks decreased serum levels of inflammatory factors, including TNF‐α and IL‐6 [33].

Furthermore, an animal experiment showed that administering 200 and 400 mg/kg of cardamom to diabetic rats improved central insulin signalling, leading to a decrease in the production of pro‐inflammatory TNF‐α and IL‐1β, a reduction in glycogen synthase kinase‐3 beta (GSK‐3β) in the hippocampus, and a decrease in the accumulation of amyloid beta (Aβ). Notably, only animals treated with 400 mg/kg of cardamom exhibited a significant increase in NR1 and NR2A mRNA expressions, which are crucial for learning and memory processes [41]. In the current study, our results showed that the concentration of TNF‐α was significantly elevated in the hippocampus tissue of the CAF diet group compared to the standard rats (*p* < 0.01). However, administering cardamom supplementation in the CAF diet group led to a significant reduction in TNF‐α levels compared to the CAF‐CARD group (120.37 ± 10.54 vs. 98 ± 12.17; *p* < 0.01; respectively).

Cardamom is also a potent anti‐anxiety plant. A study has shown that treating experimental animals with cardamom oil orally at 100 and 200 mg/kg for a month significantly improved locomotor activity. It also prevented the increase in acetylcholine esterase activity in the hippocampus and escape latency induced by aluminium neurotoxin. Additionally, cardamom exhibited significant recovery in retention latency compared to disease‐standard animals, and treatment at a dose of 200 mg/kg showed a decrease in neuronal degeneration [40].

The current study indicates that exposure to cardamom improved learning and recognition memory compared to the standard group. The cardamom‐exposed subjects exhibited a notable increase in the DI relative to the CAF group. These animals also demonstrated improved working memory and exploratory behaviour in the Y‐maze test. These results are compatible with those reported in a study conducted by Kaur et al. It was shown that epigallocatechin‐3‐gallate, a compound derived from cardamom seeds, upregulated the activity of protein kinase C in the outer membrane of the hippocampus. This compound also reduced the enzymatic conversion of glutathione peroxidase, superoxide dismutase and lipid peroxidation products while concurrently augmenting the enzymatic reaction of catalase and glutathione reductase, suggesting that cardamom may enhance memory [42].

While this study provides insights into the neuroprotective and metabolic effects of cardamom supplementation, it has several limitations. Notably, visceral adiposity (e.g., epididymal/perigonadal fat) and liver mass were not measured, which limits the assessment of systemic metabolic changes. Additionally, the cardamom extract was not chemically profiled or standardised for active constituents such as α‐terpinyl acetate and 1,8‐cineole. These omissions may affect reproducibility and mechanistic interpretation and should be addressed in future studies.

## 5 Conclusions

The current research determined that the cardamom extract of mice exhibited encouraging effects in reducing neuroinflammation. This supplementation also positively influenced memory performance and behavioural outcomes. These benefits may be attributed to the antioxidant properties of cardamom. However, the specific phytochemical constituents responsible for these effects require further investigation to understand their roles in modulating neuroinflammation and cognitive function.

## Author Contributions

Anfal AL‐Dalaeen and Nour Batarseh: methodology. Anfal AL‐Dalaeen: validation, investigation, resources and data curation, writing – original draft preparation, funding acquisition. Anfal AL‐Dalaeen, Nour Batarseh and Sally Atawneh: formal analysis. All authors have read and agreed to the published version of the manuscript.

## Ethics Statement

The animal study protocol was approved by the Institutional Review Board (or Ethics Committee) of Applied Science Private University (protocol code 2023‐PHA‐30, 6 August 2023). All animal experiments were performed according to the National Institute of Health Guide for the Care and Use of Laboratory Animals.

## Consent

The authors have nothing to report.

## Conflicts of Interest

The authors declare no conflicts of interest.

## Supporting information


**Table S1:** Composition of experimental diets.
**Table S2:** Full statistical results from two‐way ANOVA analyses.

## Data Availability

The data that support the findings of this study are available from the corresponding author upon reasonable request.

## References

[1] K. Singletary , “Cardamom Potential Health Benefits,” Nutrition Today 57 (2022): 38–49, 10.1097/NT.0000000000000507.

[2] C. Delgadillo‐Puga , I. Torre‐Villalvazo , Y. Y. Cariño‐Cervantes , et al., “Cardamom ( *Elettaria cardamomum* (L.) Maton) Seeds Intake Increases Energy Expenditure and Reduces Fat Mass in Mice by Modulating Neural Circuits That Regulate Adipose Tissue Lipolysis and Mitochondrial Oxidative Metabolism in Liver and Skeletal Muscle,” International Journal of Molecular Sciences 24 (2023): 3909, 10.3390/ijms24043909. 36835337 PMC9960522

[3] K. S. Kumar , A. Unnisa , K. Sai Sushmitha , A. Lokhande , and R. Suthakaran , “Antidepressant Activity of Cardamom Oil by Marble Burying Test in Rats,” Der Pharmacia Lettre 8 (2016): 279–282, 10.13140/RG.2.2.29097.52327.

[4] O. Asbaghi , E. Eslampour , Ž. Reiner , et al., “Effect of Green Cardamom on Lipoproteins, Glycemic Control and Anthropometric Parameters: A Meta‐Analysis of Randomized Clinical Trials,” Clinical Nutrition ESPEN 37 (2020): 24–33, 10.1016/j.clnesp.2020.03.015. 32359750

[5] S. G. Zahedi , F. Koohdani , M. Qorbani , et al., “The Effects of Green Cardamom Supplementation on Blood Pressure and Endothelium Function in Type 2 Diabetic Patients A Study Protocol for a Randomized Controlled Clinical Trial,” Medicine 99 (2020): e11005, 10.1097/MD.0000000000011005. 32358339 PMC7440108

[6] A. R. Lewis , S. Singh , and F. F. Youssef , “Cafeteria‐Diet Induced Obesity Results in Impaired Cognitive Functioning in a Rodent Model,” Heliyon 5 (2019): e01412, 10.1016/j.heliyon.2019.e01412. 30976688 PMC6441847

[7] I. C. MacEdo , L. F. Medeiros , C. Oliveira , et al., “Cafeteria Diet‐Induced Obesity Plus Chronic Stress Alter Serum Leptin Levels,” Peptides 38 (2012): 189–196, 10.1016/j.peptides.2012.08.007. 22940203

[8] A. C. Reichelt , J. Maniam , R. F. Westbrook , and M. J. Morris , “Dietary‐Induced Obesity Disrupts Trace Fear Conditioning and Decreases Hippocampal Reelin Expression,” Brain, Behavior, and Immunity 43 (2015): 68–75, 10.1016/j.bbi.2014.07.005. 25043993

[9] D. Teixeira , A. L. Cecconello , W. A. Partata , L. S. de Fraga , M. F. M. Ribeiro , and R. P. Guedes , “The Metabolic and Neuroinflammatory Changes Induced by Consuming a Cafeteria Diet Are Age‐Dependent,” Nutritional Neuroscience 22 (2019): 284–294, 10.1080/1028415X.2017.1380892. 28958196

[10] A. Shafat , B. Murray , and D. Rumsey , “Energy Density in Cafeteria Diet Induced Hyperphagia in the Rat,” Appetite 52 (2009): 34–38, 10.1016/j.appet.2008.07.004. 18680774

[11] S. K. Kanthlal , J. Joseph , B. Paul , M. Vijayakumar , and P. Uma Devi , “Antioxidant and Vasorelaxant Effects of Aqueous Extract of Large Cardamom in L‐NAME Induced Hypertensive Rats,” Clinical and Experimental Hypertension 42 (2020): 581–589, 10.1080/10641963.2020.1739699. 32202168

[12] J. Sripetchwandee , N. Chattipakorn , and S. C. Chattipakorn , “Links Between Obesity‐Induced Brain Insulin Resistance, Brain Mitochondrial Dysfunction, and Dementia,” Frontiers in Endocrinology 9 (2018): 496, 10.3389/fendo.2018.00496. 30233495 PMC6127253

[13] Z. Wohua and X. Weiming , “Glutaredoxin 2 (GRX2) Deficiency Exacerbates High Fat Diet (HFD)‐Induced Insulin Resistance, Inflammation and Mitochondrial Dysfunction in Brain Injury: A Mechanism Involving GSK‐3β,” Biomedicine & Pharmacotherapy 118 (2019): 108940, 10.1016/j.biopha.2019.108940. 31382130

[14] R. A. Arista , B. P. Priosoeryanto , and W. Nurcholis , “Profile Volatile Compounds in Essential Oils on Different Parts of Cardamom With Antioxidant Activity,” Biointerface Research in Applied Chemistry 13 (2023): 328, 10.33263/BRIAC134.328.

[15] B. S. Ahmed , “Cardamom Essential Oil‐Loaded Nanostructured Lipid Carrier Reduces Obesity and Mitigates Diabetes Mellitus in Rats Fed With High Sugar/High Fat Diet,” Natural Product Communications 19 (2024): 1934578X241286440, 10.1177/1934578X241286440.

[16] P. Ballester , B. Cerdá , R. Arcusa , A. M. García‐Muñoz , J. Marhuenda , and P. Zafrilla , “Antioxidant Activity in Extracts From Zingiberaceae Family: Cardamom, Turmeric, and Ginger,” Molecules 28 (2023): 4024, 10.3390/molecules28104024. 37241765 PMC10220638

[17] J. Hussein , M. Ashour , T. Elias , et al., “A Promising Approach for the Treatment of Experimental Alzheimer's Disease: Impact of Cardamom Essential Oil,” Egyptian Journal of Chemistry 66 (2023): 385–391, 10.21608/EJCHEM.2023.210897.7974.

[18] J. Carillon , C. Romain , G. Bardy , et al., “Cafeteria Diet Induces Obesity and Insulin Resistance Associated With Oxidative Stress but Not With Inflammation: Improvement by Dietary Supplementation With a Melon Superoxide Dismutase,” Free Radical Biology & Medicine 65 (2013): 254–261, 10.1016/j.freeradbiomed.2013.06.022. 23792771

[19] C. M. do Nascimento , F. A. Felipetti , T. Cassol , et al., “Evaluation of Bone Tissue After Cafeteria‐Diet‐Induced Obesity and Periodontitis in Rats,” Journal of Endocrinology and Metabolism 5 (2015): 172–178, 10.14740/jem248w.

[20] U. Tahir and M. R. Khan , Cardamom ( *Elettaria cardamomum* ): Production, Processing and Properties (Springer Nature, 2023), 10.1007/978-3-031-35426-7.

[21] S. Andrikopoulos , A. R. Blair , N. Deluca , B. C. Fam , and J. Proietto , “Evaluating the Glucose Tolerance Test in Mice,” American Journal of Physiology. Endocrinology and Metabolism 295 (2008): E1323–E1332, 10.1152/ajpendo.90617.2008. 18812462

[22] A. Ferreira , J. P. Castro , J. P. Andrade , M. Dulce Madeira , and A. Cardoso , “Cafeteria‐Diet Effects on Cognitive Functions, Anxiety, Fear Response and Neurogenesis in the Juvenile Rat,” Neurobiology of Learning and Memory 155 (2018): 197–207, 10.1016/j.nlm.2018.07.014. 30075193

[23] M. H. Sivakumaran , A. K. Mackenzie , I. R. Callan , J. A. Ainge , and A. R. O'Connor , “The Discrimination Ratio Derived From Novel Object Recognition Tasks as a Measure of Recognition Memory Sensitivity, Not Bias,” Scientific Reports 8 (2018): 11579, 10.1038/s41598-018-30030-7. 30069031 PMC6070491

[24] E. Prieur and N. Jadavji , “Assessing Spatial Working Memory Using the Spontaneous Alternation Y‐Maze Test in Aged Male Mice,” Bio‐Protocol 9 (2019): 1–10, 10.21769/bioprotoc.3162. PMC7854095 33654968

[25] M. Angoa‐pérez , M. J. Kane , D. I. Briggs , D. M. Francescutti , and D. M. Kuhn , “Marble Burying and Nestlet Shredding as Tests of Repetitive,” Compulsive‐Like Behaviors in Mice 24 (2013): 50978, 10.3791/50978. PMC4108161 24429507

[26] S. Parham , A. Z. Kharazi , H. R. Bakhsheshi‐Rad , et al., “Antioxidant, Antimicrobial and Antiviral Properties of Herbal Materials,” Antioxidants 9 (2020): 1–36, 10.3390/antiox9121309. PMC7767362 33371338

[27] M. Riaz , R. Khalid , M. Afzal , et al., “Phytobioactive Compounds as Therapeutic Agents for Human Diseases: A Review,” Food Science & Nutrition 11 (2023): 2500–2529, 10.1002/fsn3.3308. 37324906 PMC10261751

[28] R. Yahyazadeh , M. G. Rahbardar , B. M. Razavi , G. Karimi , and H. Hosseinzadeh , “The Effect of Elettaria Cardamomum (Cardamom) on the Metabolic Syndrome: Narrative Review,” Iranian Journal of Basic Medical Sciences 24 (2021): 1462–1469, 10.22038/IJBMS.2021.54417.12228. 35317114 PMC8917848

[29] Y. Duan , L. Zeng , C. Zheng , et al., “Inflammatory Links Between High Fat Diets and Diseases,” Frontiers in Immunology 9 (2018): 1–10, 10.3389/fimmu.2018.02649. 30483273 PMC6243058

[30] R. S. M. Labban , H. Alfawaz , A. T. Almnaizel , et al., “High‐Fat Diet‐Induced Obesity and Impairment of Brain Neurotransmitter Pool,” Translational Neuroscience 11 (2020): 147–160, 10.1515/tnsci-2020-0099. 33312720 PMC7705990

[31] N. M. Vega‐Rivera , E. Estrada‐Camarena , G. Azpilcueta‐Morales , et al., “Chronic Variable Stress and Cafeteria Diet Combination Exacerbate Microglia and c‐Fos Activation but Not Experimental Anxiety or Depression in a Menopause Model,” International Journal of Molecular Sciences 25 (2024): 1455, 10.3390/ijms25031455. 38338735 PMC10855226

[32] N. H. Phelps , R. K. Singleton , B. Zhou , et al., “Worldwide Trends in Underweight and Obesity From 1990 to 2022: A Pooled Analysis of 3663 Population‐Representative Studies With 222 Million Children, Adolescents, and Adults,” Lancet 403 (2024): 1027–1050, 10.1016/S0140-6736(23)02750-2. 38432237 PMC7615769

[33] S. Cheshmeh , N. Elahi , M. Ghayyem , et al., “Effect of Green Cardamom on the Expression of Genes Implicated in Obesity and Diabetes Among Obese Women With Polycystic Ovary Syndrome: A Double Blind Randomized Controlled Trial,” Genes & Nutrition 17 (2022): 17, 10.1186/s12263-022-00719-6. 36522620 PMC9753872

[34] G. M. Abu‐Taweel , “Cardamom ( *Elettaria cardamomum* ) Perinatal Exposure Effects on the Development, Behavior and Biochemical Parameters in Mice Offspring,” Saudi Journal of Biological Sciences 25 (2018): 186–193, 10.1016/j.sjbs.2017.08.012. 29379379 PMC5775110

[35] M. M. Rahman , M. N. Alam , A. Ulla , et al., “Cardamom Powder Supplementation Prevents Obesity, Improves Glucose Intolerance, Inflammation and Oxidative Stress in Liver of High Carbohydrate High Fat Diet Induced Obese Rats,” Lipids in Health and Disease 16 (2017): 1–12, 10.1186/s12944-017-0539-x. 28806968 PMC5557534

[36] H. Winarsi , N. D. Sasongko , A. Purwanto , and I. Nuraeni , “Effect of Cardamom Leaves Extract as Antidiabetic, Weight Lost and Hypocholesterolemic to Alloxan‐Induced Sprague Dawley Diabetic Rats,” International Food Research Journal 21 (2014): 2253–2261.

[37] W. J. Adeyemi , L. A. Olayaki , T. A. Abdussalam , et al., “Omega‐3 Fatty Comparative Evaluation of the Pharmacological Value of Virgin Coconut Oil, Omega 3 Fatty Acids, and Orlistat in Experimental Study on Obesity With Normo/Hyper‐Lipidaemic Diet,” PharmaNutrition 13 (2020): 100192, 10.1016/j.phanu.2020.100192.

[38] N. Shakeeb , P. Varkey , A. Hynse , and A. Mandlecha , “Anti‐Inflammatory Response of Cardamom Extract and Prediction of Therapeutic Window in COVID‐19 Patients by Assessing Inflammatory Markers Using RT‐PCR,” Inflammopharmacology 30 (2022): 883–894, 10.1007/s10787-022-00951-x. 35320494 PMC8941370

[39] S. Sreedharan , V. Nair , and L. Cisneros‐zevallos , “Protective Role of Phenolic Compounds From Whole Cardamom,” Nutrientes 15 (2023): 2965. 10.3390/nu15132965 PMC10346154 37447289

[40] S. T. Auti and Y. A. Kulkarni , “Neuroprotective Effect of Cardamom Oil Against Aluminum Induced Neurotoxicity in Rats,” Frontiers in Neurology 10 (2019): 1–17, 10.3389/fneur.2019.00399. 31114535 PMC6502995

[41] A. A. Gomaa , R. M. Makboul , M. A. El‐Mokhtar , E. A. Abdel‐Rahman , I. A. Ahmed , and M. A. Nicola , “Terpenoid‐Rich *Elettaria cardamomum* Extract Prevents Alzheimer‐Like Alterations Induced in Diabetic Rats via Inhibition of GSK3β Activity, Oxidative Stress and Pro‐Inflammatory Cytokines,” Cytokine 113 (2019): 405–416, 10.1016/j.cyto.2018.10.017. 30539783

[42] S. K. Bhathal , H. Kaur , K. Bains , and A. K. Mahal , “Assessing Intake and Consumption Level of Spices Among Urban and Rural Households of Ludhiana District of Punjab, India,” Nutrition Journal 19 (2020): 1–12, 10.1186/s12937-020-00639-4. 33158443 PMC7648309

