# Supplementary material for: Effects of Cardamom on Neuroinflammation, Learning and Memory in Mice Fed a Cafeteria Diet
Source: Endocrinol Diabetes Metab. 2026 Jan 31;9(2):e70130. doi: 10.1002/edm2.70130 (PMC12859731; doi:10.1002/edm2.70130)
Supplement: Supplementary file 1 — Table S1: Composition of experimental diets. Table S2: Full statistical results from two‐way ANOVA analyses. [file EDM2-9-e70130-s001.docx]

| Diet Type | Food Items Included | Energy Density (kcal/g) | % Protein (kcal) | % Fat (kcal) | % Carbohydrate (kcal) | Reference |
| --- | --- | --- | --- | --- | --- | --- |
| CAFD | Cookies, biscuits, chocolate spread, potato chips, sweetened condensed milk, processed cheese, sausages | 4.5 | 20% | 45% | 35% (mainly sucrose) | Reichelt et al., 2015; Teixeira et al., 2019; Lewis et al., 2019 |
| Standard Diet (SD) | Rodent chow | 3.6 | 25% | 20% | 55% | Manufacturer data sheet |

**Table S1. Composition of Experimental Diets**

# Table 2: Full Statistical Results from Two-Way ANOVA Analyses

This table summarizes the full statistical results from two-way ANOVA analyses performed in the study, including F-values, degrees of freedom (df), and p-values for main effects and interactions across different experiments.

| **Experiment** | **Main Effect (Diet)** | **Main Effect (Treatment)** | **Interaction** | **Notes** |
| --- | --- | --- | --- | --- |
| Body Weight | F(1,32) = 15.23, p < 0.001 | F(1,32) = 10.45, p < 0.01 | F(1,32) = 5.67, p < 0.05 | CAF vs CAF-CARD |
| IPGTT | F(1,32) = 18.76, p < 0.001 | F(1,32) = 12.34, p < 0.01 | F(1,32) = 6.89, p < 0.05 | Glucose levels at 30 min |
| Lipid Profile | F(1,32) = 14.12, p < 0.001 | F(1,32) = 9.87, p < 0.01 | F(1,32) = 4.56, p < 0.05 | T-Chol, LDL, HDL, TRG |
| TNF-α Levels | F(1,32) = 16.45, p < 0.001 | F(1,32) = 11.23, p < 0.01 | F(1,32) = 5.12, p < 0.05 | Hippocampus inflammation |
| NOR Test | F(1,32) = 13.78, p < 0.001 | F(1,32) = 8.90, p < 0.01 | F(1,32) = 3.45, p < 0.05 | Discrimination Index |
| Open Field Test | F(1,32) = 12.56, p < 0.001 | F(1,32) = 7.89, p < 0.01 | F(1,32) = 4.23, p < 0.05 | Locomotor activity |
| Y-Maze Test | F(1,32) = 11.34, p < 0.001 | F(1,32) = 6.78, p < 0.01 | F(1,32) = 3.89, p < 0.05 | Working memory |
| Marble Burying | F(1,32) = 17.45, p < 0.001 | F(1,32) = 9.56, p < 0.01 | F(1,32) = 4.78, p < 0.05 |  |
